# Biomimetic Design of Underwater Adhesives Based on Tea Polyphenol-Modified Gelatin

**DOI:** 10.3390/biomimetics10030149

**Published:** 2025-02-28

**Authors:** Ziwei Wu, Zhipeng Li, Yixiao Li, Haoyu Wang, Jiang Yue, Tieling Xing

**Affiliations:** 1 National Engineering Laboratory for Modern Silk, College of Textile and Clothing Engineering, Soochow University, Suzhou 215123, China; 20244215044@stu.suda.edu.cn (Z.W.); 20224215010@stu.suda.edu.cn (Z.L.); 20244215039@stu.suda.edu.cn (Y.L.); 20245215079@stu.suda.edu.cn (H.W.); 2 China National Textile and Apparel Council Key Laboratory of Natural Dyes, College of Textile and Clothing Engineering, Soochow University, Suzhou 215123, China; 3 School of Medicine, Shanghai Jiaotong University, Shanghai 200127, China

**Keywords:** gelatin, tea polyphenols, gallic acid, biomimetic design, underwater adhesives

## Abstract

Although many tissue adhesives with good biocompatibility are currently available, their lack of wet adhesion capacity significantly hinders their clinical application. Therefore, further development and exploration of new medical adhesives are necessary. Inspired by the adhesion mechanism of marine mussels, through modifying gelatin protein with gallic acid (GA) for wet adhesion and cross-linking gelatin (Gel) molecular chains with tea polyphenols (TP), the adhesive TP-GA/Gel was developed. The adhesive exhibited an adhesion strength of up to 130.47 kPa to porcine skin tissues and maintained a high adhesion state in various aqueous environments, demonstrating excellent and reproducible adhesion. Additionally, TP-GA/Gel possessed outstanding antimicrobial, antioxidant, and biocompatibility properties. In an in vivo wound healing study with SD rats, the wound area treated with TP-GA/Gel adhesive decreased from 10.3 mm^2^ to 0.9 mm^2^ after 15 days, promoting effective and scarless wound healing. These results highlight the promising clinical potential of TP-GA/Gel as a medical adhesive.

## 1. Introduction

In modern medicine, wound healing and tissue repair are critical aspects of patient care [1,2,3]. Traditional wound closure methods, such as sutures and wound closure patches, have been widely used for a long time. However, they have certain limitations. Sutures require a high level of surgical skill, involve prolonged and precise manipulation, and may cause tissue trauma, potentially leading to scarring and increased risk of infection [4,5]. Additionally, sutures can cause patient discomfort and may not be suitable for fragile or small sized wounds. Wound closure patches, while effective for small, non-wet, and dry wounds, may cause scarring and secondary pain. However, they are less effective for irregularly shaped wounds or those with more severe injuries [6]. In 1959, rapid adhesives based on α-cyano-methyl acrylate (CA) were introduced in the United States, revolutionizing surgical procedures by shifting from suturing to direct bonding. Despite their many advantages, CA-based adhesives have limitations, including exothermic polymerization reactions, the release of toxic monomers, low viscosity, and weak mechanical properties, which have hindered their widespread clinical use [7]. Therefore, the development of multifunctional medical adhesives that can effectively close wounds while promoting scarless healing remains a crucial goal in modern medicine [8].

Gelatin (Gel) is a macromolecular protein of animal origin, typically obtained through the partial hydrolysis of collagen [9]. In physiological environments, Gel exhibits good biocompatibility, degradability, and low antigenicity, making it attractive for applications in tissue engineering, wound dressings, and surgical adhesives. Gel provides numerous adhesion sites due to its molecular structure, which contains functional groups, such as -COOH, -NH_2_, and -OH, that can form hydrogen bonds. However, Gel lacks strong bioadhesion properties. Given the unique properties and structural diversity of Gel, there has been growing interest in enhancing its bioadhesion properties to improve its application in the pharmaceutical field. Several strategies have been proposed to achieve this, including the formation of Gel complexes and chemical modification to enhance its tissue adhesion. Common modifications to Gel include amination, cross-linking, and phenol coupling. Matsuda et al. [10] developed strong bioadhesive Gel adhesives by treating Gel with glutaraldehyde to introduce free aldehyde groups. These aldehyde groups form covalent bonds with amino groups on the tissue surface through a Schiff base reaction, significantly improving adhesion strength. Cohen et al. [11] prepared a Gel and alginate based tissue adhesive by cross-linking with EDC (1-(3-dimethylaminopropyl)-3-ethylcarbodiimide). During cross-linking, EDC binds to the carboxyl group (from either Gel or alginate) and forms a transient o-isoacylurea derivative. This derivative undergoes a nucleophilic attack by the amino group, resulting in the formation of an amide bond. However, the biosafety of cross-linking agents like glutaraldehyde and EDC requires further investigation. Therefore, it is crucial to develop non-toxic crosslinkers that preserve the adhesive properties of Gel.

Marine mussels possess a unique adhesive protein that enables them to adhere firmly to rocks, even in the extreme conditions of the ocean [12,13]. This extraordinary natural phenomenon has inspired the development of new adhesives by mimicking the composition of this protein. The mussel protein secreted by marine mussels is rich in 3,4-dihydroxyphenylalanine (L-DOPA), a component that plays a crucial role in the adhesion process [14]. For instance, the aromatic ring in L-DOPA can stack with other aromatic groups through π-π interactions, while the catechol groups can form coordination bonds with metals, and the hydroxyl groups can form hydrogen bonds with polar molecules on the substrate, all of which enhance adhesion [15]. In an oxidizing environment, L-DOPA can be oxidized to form quinone groups, which can then form covalent bonds with other molecules, resulting in chemical cross-linking and further enhancing adhesion [16].

Gallic acid (GA) is a common naturally occurring phenolic compound believed to possess a variety of biological activities, including antioxidant, antimicrobial, and anti-inflammatory properties [17]. GA contains three adjacent hydroxyl groups on the benzene ring, a novel functional group capable of mimicking the adhesion of mussels. Haitai et al. [18] developed a hyaluronic acid tissue adhesive functionalized with GA, which showed good viscoelasticity, stable swelling behavior, and some antioxidant activity.

Tea polyphenols (TPs) are a class of bioactive compounds widely found in tea, known for their various biological activities, such as antioxidant, anti-inflammatory, antibacterial, and antitumor properties, which have been shown to have a positive impact on human health [19]. Their antioxidant properties can help scavenge free radicals and slow oxidative cell damage. TP belongs to the class of macromolecular polyphenolic compounds with multiple benzene rings and hydroxyl groups, which can act as cross-linking agents within macromolecular polymers. Meanwhile, the phenolic hydroxyl groups exhibit strong electrophilicity and nucleophilicity, allowing them to react with the functional groups on the surface of biological materials or within tissues.

**Figure 1:**
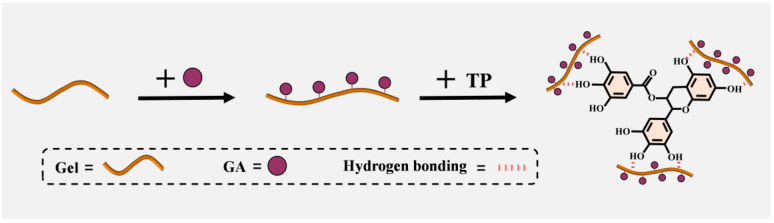
Schematic of TP-GA/Gel adhesive preparation process.

In this study, a biomimetic adhesive was developed based on the adhesion mechanism observed in mussels. Gel was used as the adhesive matrix, and the concept of the wet adhesion modification of protein molecular chains by small molecule polyphenols, followed by the further cross-linking of modified protein chains with large molecule polyphenols, was adopted. This approach led to the proposal of a polyphenol-modified gelatin-protein-based adhesive for medical use. Polyphenols contain benzene ring structures that can undergo π-π stacking interactions with Gel. TPs contribute to the adhesive formation through the hydrophobicity of the aromatic rings in their molecular structure, followed by multiple hydrogen bonding. Additionally, the oxidation of phenolic hydroxyl groups to quinones within GA promotes further cross-linking [20]. A flowchart illustrating the preparation of the designed adhesive is shown in Figure 1.

## 2. Materials and Methods

### 2.1. Materials

Gelatin (Gel), gallic acid (GA), and tea polyphenols (TP) were provided by Shanghai Yuanye Biotechnology Co., Ltd. (Shanghai, China), with the Gel being of biotechnology grade. Urea was purchased from Aladdin Industrial Corporation (Shanghai, China). Fresh porcine skin was sourced from the market. Distillation−distillation water (ddH_2_O) was used without further purification in all the experiments.

### 2.2. Preparation of TP-GA/Gel

The adhesive was prepared using a blending method. First, a 10 wt% Gel aqueous solution was prepared, and 0.15 g of GA was added to 10 mL of the Gel solution. The mixture was then purged with nitrogen and slow stirring in the dark for 6 h. Then, a 10 wt% TP aqueous solution was prepared, and 10 mL of TP solution was added to the prepared GA/Gel solution. The two solutions were blended to form a viscous mixture. Finally, the TP-GA/Gel adhesive was washed by deionized water to remove residual surface impurities.

### 2.3. Structural Characterization

An ultraviolet−visible spectrophotometer (UV–vis, UV1900, Puxi, Beijing, China) was utilized to analyze the samples. The Fourier transform infrared (FT-IR) spectra of the adhesive were obtained using an FT-IR spectrometer (Nicolet 5700, Thermo Fisher Scientific, Waltham, MA, USA), scanning in the range of 4000 to 500 cm^−1^. To observe the microstructure and analyze surface elements, the freeze-dried adhesive was investigated using a scanning electron microscope (FESEM, Hitachi S-8100, Hitachi Limited, Tokyo, Japan) after being coated with a thin layer of gold.

### 2.4. Solid Content Test

The mass of each TP-GA/Gel sample was measured both before ($m_{1}$) and after ($m_{2}$) lyophilization. The SC was determined using Equation (1). Each sample was tested three times, and the average value was calculated.

(1) $$SC\left(\%\right)=\frac{m_{2}}{m_{1}}\times 100\%$$

### 2.5. Rheological Properties

The urea-containing adhesive, referred to as TP-GA/Gel-urea, was first obtained by immersing it in a 1 mol/L urea solution for 24 h. The rheological properties of TP-GA/Gel and TP-GA/Gel-urea were then analyzed. Frequency sweep measurements were performed using a rheometer (AR2000 DHR-2, TA Instruments, New Castle, DE, USA) with a 20 mm parallel plate with a 1 mm gap. The viscoelastic behavior of the adhesives was evaluated by measuring the storage modulus (G′) and loss modulus (G″) in frequency sweep mode over a frequency range of 0.1–10 Hz.

### 2.6. Adhesion Testing

Adhesion testing was conducted to evaluate the adhesion strength of the TP-GA/Gel adhesive. The lap shear test followed a modified ASTM Standard F2255 and previously established methods [21]. All the test substrates, including wood, steel, glass, porcine skin, polytetrafluoroethylene (PTFE), and polyethylene (PE), were cut to 1 cm × 3 cm, with the adhesive applied to a 1 cm × 1 cm area. The adhesion performance of TP-GA/Gel on different substrates was evaluated. To assess adhesive strength, the adhesive was applied to different substrates and immersed in water for 30 min. To test the adhesion strength of the adhesive to porcine skin after different adhesion times, the porcine skin was adhered in water for different periods, including 1 min, 10 min, 30 min, 1 h, 6 h, 12 h, 18 h, and 24 h. Additionally, to evaluate the adhesion strength of the adhesive to porcine skin in different aqueous environments, the adhesive was applied to porcine skin and then immersed in various aqueous solutions (distilled water, DMEM, PBS, pH 6, and pH 8) for 30 min. To quantitatively evaluate the reusable bonding properties, two pieces of porcine skin were bonded underwater using the TP-GA/Gel adhesive and allowed to solidify in water for 30 min. Cyanoacrylate adhesive was used as a control. A lap shear test was then performed until the bond failed, and the bonding capacity was recorded. The bonded area, initially coated with adhesive, was manually pressed underwater for 3–5 s before being reattached and quickly tested for repeated adhesion strength. A total of 10 adhesion tests were conducted.

### 2.7. Antibacterial Properties Testing

The antimicrobial properties of TP-GA/Gel adhesive were evaluated using Gram-negative *Escherichia coli* (*E. coli*) and Gram-positive *Staphylococcus aureus* (*S. aureus*). A single colony from strains of 3–10 generations was selected and inoculated into 20 mL of nutrient solution, followed by incubation at 37 °C for 18–24 h on a shaker. Subsequently, 1 mL of the initial bacterial culture was taken and diluted to a certain concentration in nutrient solution and phosphate buffer solution (PBS). Approximately 0.2 g of the TP-GA/Gel adhesive was soaked in the diluted broth, allowing the antimicrobial substances released by TP-GA/Gel to infuse the broth. This antimicrobial broth was then used for further experiments. Bacteria cultured without the adhesive served as the control group. The bacterial solution was incubated on an oscillating platform at 24 °C for 18–24 h. Then, 1 mL from each sample set was extracted and diluted to the appropriate concentration using the 10-fold dilution method, followed by further incubation with shaking for 18–24 h. Moreover, two parallel samples were prepared for each experimental set. The viability rate was calculated using Equation (2):

(2) $$Y(\%)=\frac{Q_{t}}{W_{t}}\times 100\%$$

where *Y* is the bacterial viability, $W_{t}$ is the number of colonies in the blank petri dish after 18 h of oscillatory contact, and $Q_{t}$ is the number of colonies in the TP-GA/Gel petri dish after 18 h of oscillatory incubation.

### 2.8. Antioxidant PropertiesTesting

2,2-diphenyl-1-picrylhydrazyl (DPPH) was used to simulate free radicals, and a DPPH solution was prepared using methanol as a solvent at a concentration of 0.04 mg mL^−1^. Subsequently, 3 mL of DPPH solution was mixed with 50 mg of TP-GA/Gel adhesive and placed in an oven at 37 °C, protected from light, for 90 min to allow the reaction to proceed. The pure DPPH solution was used as the control. The absorbance of the DPPH solution was measured by UV spectrometry at 516 nm, using methanol as the baseline. The DPPH radical scavenging efficiency was then calculated using Equation (3):

(3) $$DPPH\;scavenging(\%)=\frac{A_{B}-A_{S}}{A_{B}}\times 100\%$$

where $A_{B}$ represents the DPPH absorbance without the addition of TP-GA/Gel adhesive (blank group), and $A_{s}$ represents the DPPH absorbance after the addition of TP-GA/Gel adhesive (experimental group).

### 2.9. Cytotoxicity Experiments

The cytotoxicity of the adhesives was assessed using the CCK-8 (Cell Counting Kit 8, Kesaien, Suzhou, China) assay with L929 cells (mouse fibroblasts obtained from the Shanghai Cell Bank of the Chinese Academy of Sciences) directly exposed to the adhesive leachate. The absorbance of the medium was then measured at 450 nm using an enzyme marker. The relative cell viability was calculated using Equation (4):

(4) $$Cell\;viability(\%)=\frac{{Abs}_{adhesive}}{{Abs}_{control}}\times 100\%$$

where ${Abs}_{adhesive}$ represents the absorbance of the cells after incubation with the adhesive extract, and ${Abs}_{control}$ represents the absorbance of the blank control group culture.

### 2.10. Wound Healing Properties

To evaluate the wound healing properties of TP-GA/Gel in vivo, animal experiments were performed using male Sprague–Dawley (SD) rats (240–280 g) obtained from the Experimental Animal Centre of Soochow University. The rats were anaesthetized with a 10 wt% chloral hydrate solution and secured on a wooden board on the operating table. Their backs were shaved, and the exposed skin was disinfected with alcohol. A 1.0 cm-long wound was then created on the back of each rat using a medical scalpel. In the experimental group, approximately 0.5 g of adhesive was applied to the wound for adhesion, while the control group was left untreated. The wound area was monitored and photographed on days 0, 5, 10, and 15. Image J software (v1.8.0) was used to analyze and calculate the wound area, and the data were recorded.

## 3. Results and Discussion

### 3.1. Characterization of the Adhesive

**Figure 2:**
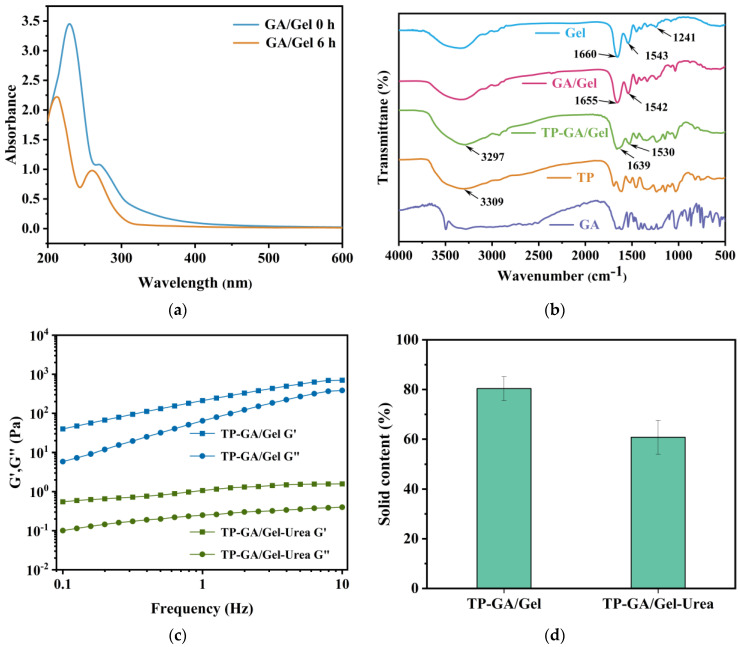
(**a**) UV spectra of GA/Gel 0 h and GA/Gel 6 h; (**b**) FT-IR spectra of pure Gel, GA/Gel, TP-GA/Gel, TP, and GA; (**c**) rheological analysis of TP-GA/Gel and TP-GA/Gel-urea; and (**d**) SC of TP-GA/Gel and TP-GA/Gel-urea.

UV-visible spectroscopic analysis is an effective method for examining phenolic structural changes during the oxidative polymerization of phenols [22]. Figure 2a shows the UV-visible spectra of GA/Gel 0 h and GA/Gel 6 h. Compared to GA/Gel 0 h, the spectrum of GA/Gel 6 h exhibits a clear broad band between 250 nm and 400 nm, corresponding to the absorption range of quinone. This indicates that quinone was formed due to the partial oxidative polymerization of GA in Gel solution. Quinone may further undergo cross-linking reactions between GA and Gel molecules [23]. As shown in Figure 2b, Gel displays characteristic infrared absorption peaks at 1660 cm^−1^ (amide I, C=O stretching), 1543 cm^−1^ (amide II, C=O stretching, N-H bending, and C=N stretching vibration) and 1241 cm^−1^ [24]. In contrast, the amide I and amide II peaks in GA/Gel shift to 1655 cm^−1^ and 1542 cm^−1^, respectively, indicating the occurrence of a Schiff base reaction and the rearrangement of the Gel triple-helical structure with enhanced internal hydrogen bonding [25]. When the GA/Gel solution was mixed with the TP solution, TP initially interacted with Gel proteins through hydrophobic interactions between the aromatic rings in its molecular structure. This was followed by multiple hydrogen bonding interactions, ultimately forming the TP-GA/Gel adhesive. Compared to Gel, the amide I absorption peak of TP-GA/Gel was 1639 cm^−1^, and the amide II absorption peak was 1530 cm^−1^, indicating a redshift in both bands, which proved the formation of hydrogen bonding between TP and GA/Gel. Additionally, the phenolic hydroxyl (-OH) peak shifted from 3309 cm^−1^ in TP to 3297 cm^−1^ in TP-GA/Gel, further supporting the presence of strong hydrogen bonding within the adhesive [26].

The presence of hydrogen bonding was verified using FTIR spectroscopy. To validate the existence of the physical cross-linking network between GA/Gel and TP, the TP- GA/Gel adhesive was exposed to urea, a widely known hydrogen-bond disruptor. The rheological analysis of the adhesives showed that the energy storage modulus (G′) of both TP-GA/Gel and TP-GA/Gel-Urea, before and after urea treatment, was greater than their respective loss modulus (G″), suggesting that both samples maintained a better viscoelastic solid state morphology (Figure 2c). The energy storage modulus and loss modulus of TP-GA/Gel increased with frequency, indicating that the crosslinked network structure of the adhesive exhibits good viscoelasticity and can store or dissipate energy at different frequencies to maintain structural stability. After urea treatment, the energy storage modulus and loss modulus of TP-GA/Gel-Urea were lower than those of TP-GA/Gel, and the changes in the energy storage modulus and loss modulus of TP-GA/Gel-Urea were smaller with an increasing frequency. This indicated that urea, as a hydrogen-bond disruptor, partially disrupted the hydrogen-bonding interactions in the TP-GA/Gel adhesive, affecting the dense network structure to some extent. However, some cross-linking structures (e.g., π-π stacking, cation-π interactions, Michael addition, etc.) were not affected by urea, allowing 60.87% of the solid content to remain intact (Figure 2d).

### 3.2. Adhesion Properties

**Figure 3:**
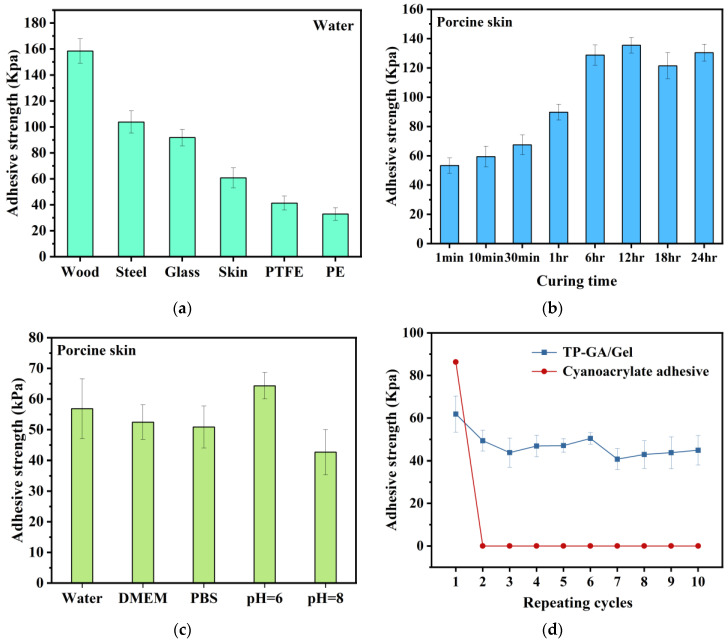
(**a**) Adhesion strength of TP-GA/Gel adhesive to different substrates underwater; (**b**) adhesion strength of TP-GA/Gel to porcine skin tissues underwater after different time of adhesion; (**c**) adhesion strength of TP-GA/Gel adhesive to porcine skin in different water environments; and (**d**) reusable adhesion strength of TP-GA/Gel.

The adhesion of TP-GA/Gel is universal; in addition to adhering to porcine skin tissue, it also exhibits a certain degree of adhesion to other substrates (Figure 3a). As shown in the figure, the adhesive strength is approximately 158.42 kPa for wood chips, 103.71 kPa for steel chips, 91.76 kPa for glass, 60.79 kPa for porcine skin, 41.31 kPa for PTFE, and 32.87 kPa for PE. Lignin in wood chips is a complex phenolic compound that contains various active functional groups, such as phenolic hydroxyl, alcohol hydroxyl, and an aromatic group [27]. This allows the TP-GA/Gel adhesive to easily form hydrogen bonds and π-π stacking interactions with wood chips. Additionally, the high surface roughness of wood chips results in high adhesion strength. The adhesion to steel chips is primarily due to strong metal–ligand bonding between the phenolic hydroxyl groups. The adhesive’s bonding to glass is mainly due to hydrogen bonding with oxygen atoms in SiO_2_. TP-GA/Gel adheres to porcine skin through hydrogen bonding, van der Waals forces, Michael addition, and other interactions with the amino, carboxyl, and sulfhydryl groups in porcine skin tissue.

In the aqueous environment, the adhesive strength to porcine skin tissue showed a gradual increase with bonding time and stabilized after 6 h (Figure 3b). The adhesion strength reached 53.32 kPa at 1 min, due to the polyphenol structure in the adhesive and the strong hydrogen bonding between the amino and carboxyl groups in Gel and the functional groups on the porcine skin tissue. After 6 h of adhesion, the adhesion strength of the adhesive to porcine skin reached its maximum value and maintained stable at approximately 130 kPa. This increase is due to the covalent cross-linking of the polyphenol groups within the adhesive through partial oxidation over time, which significantly enhances the cohesive strength. Additionally, the quinone groups can covalently cross-link with the nucleophilic groups (-NH_2_ and -SH) on the porcine skin, further strengthening the interaction between the adhesive and the porcine skin tissue.

During the simulation of the body fluid environment, the adhesion strengths in DMEM solution (52.47 kPa) and PBS solution (50.86 kPa) were found to be similar to that in distilled water (56.84 kPa), suggesting that the amino acids and glucose in the DMEM solution, as well as the Na^+^, Cl^−^, and ${PO}_{4}^{3^{-}}$ in the PBS solution, had minimal effect on the TP-GA/Gel adhesive. In an acid-base environment, the adhesion strength of the adhesive at pH 6 (64.33 kPa) was significantly higher than at pH 8 (42.66 kPa). This suggests that phenolic hydroxyl groups play an important role in the early stage of adhesion. Under alkaline conditions, some of the phenolic hydroxyl groups interact with OH^−^, reducing the number of available phenolic hydroxyl groups that can reach the surface of the porcine skin tissue, thereby decreasing the adhesion strength (Figure 3c).

To explore the reusable bonding performance of TP-GA/Gel in an aqueous environment, we conducted cyclic adhesion tests of TP-GA/Gel adhesive and cyanoacrylate on porcine skin tissue underwater (Figure 3d). The initial adhesion strength of TP-GA/Gel adhesive to porcine skin tissue was 61.87 kPa, and while the adhesion strength decreased in the subsequent cyclic tests, it remained above 40 kPa. In contrast, cyanoacrylate lost its adhesion in the later cyclic tests after an initial adhesion strength of 86.30 kPa. This indicates that the underwater reusability of TP-GA/Gel adhesive is superior to that of cyanoacrylate, which exhibits irreversible adhesion underwater.

**Figure 4:**
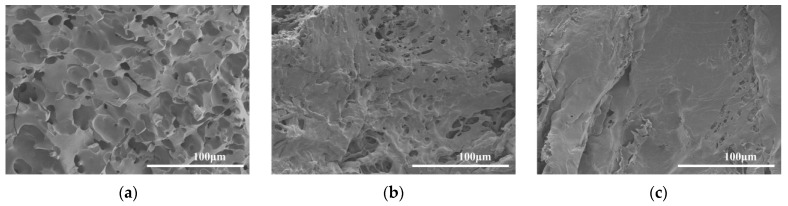
SEM images of TP-GA/Gel adhesive after 10 min (**a**), 6 h (**b**), and 12 h (**c**) of adhesion of TP-GA/Gel adhesive to porcine skin tissue under water.

The morphology of the TP-GA/Gel adhesive was analyzed after 10 min, 6 h, and 12 h of underwater adhesion to porcine skin using SEM. A gradual decrease in the number of pores within the TP-GA/Gel adhesive was observed (Figure 4). Initially, some of the phenolic hydroxyl groups of the adhesive formed bonds with nucleophilic groups (-NH and -SH) on the porcine skin tissue, while the remaining phenolic hydroxyl groups not involved in anchoring were gradually oxidized to quinones. As the curing process continued, Gel was further cross-linked with quinone, resulting in a more stable and compact molecular structure within the adhesive, which led to a reduction in pore size and pore shrinkage.

### 3.3. Antibacterial Properties

**Figure 5:**
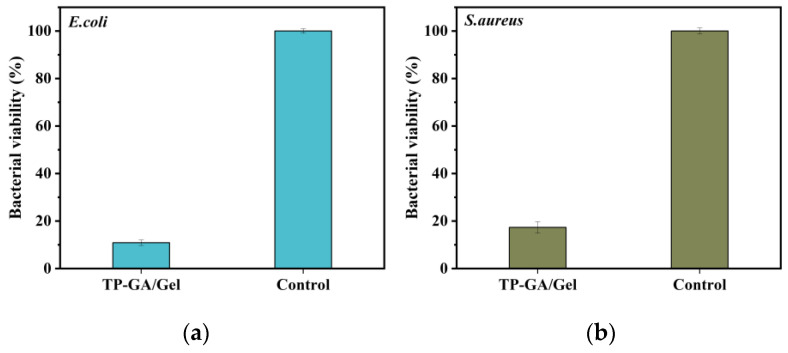
Bacterial survival of TP-GA/Gel adhesive against *E. coli* (**a**) and *S. aureus* (**b**).

The antimicrobial properties of the TP-GA/Gel adhesive were tested using *Escherichia coli* (*E. coli*) and *Staphylococcus aureus* (*S. aureus*). As shown in Figure S1, both *E. coli* and *S. aureus* in the control group grew across the whole petri dish, whereas the number of colonies decreased to different degrees after the addition of the TP-GA/Gel adhesive during the culture process. Figure 5a,b show that the survival rate of *E. coli* was 10.87%, while that of *S. aureus* was 17.33%. The GA and TP components in the TP-GA/Gel adhesive act as natural antibacterial agents [28], inhibiting bacterial growth and reproduction by interacting with lipids and proteins on bacterial cell membranes and disrupting bacterial metabolic processes. These antimicrobial experiments illustrate that TP-GA/Gel adhesive effectively inhibits both *E. coli* and *S. aureus*.

**Figure 6:**
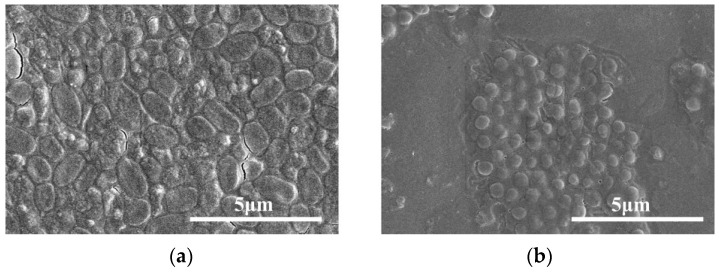
SEM images of *E. coli* (**a**) and *S. aureus* (**b**) after TP-GA/Gel adhesive treatment.

From the SEM images of *E. coli* and *S. aureus* in Figure S2, it was observed that the normal-growing *E. coli* (a) and *S. aureus* (b) maintained intact shapes, appearing as regular rods or spheres. However, as shown in Figure 6, *E. coli* (a) and *S. aureus* (b) treated with the TP-GA/Gel adhesive exhibited structural damage with rod-shaped contraction or spherical folds, indicating that TP-GA/Gel adhesive disrupts the normal growth of both bacteria.

### 3.4. Antioxidant Properties

**Figure 7:**
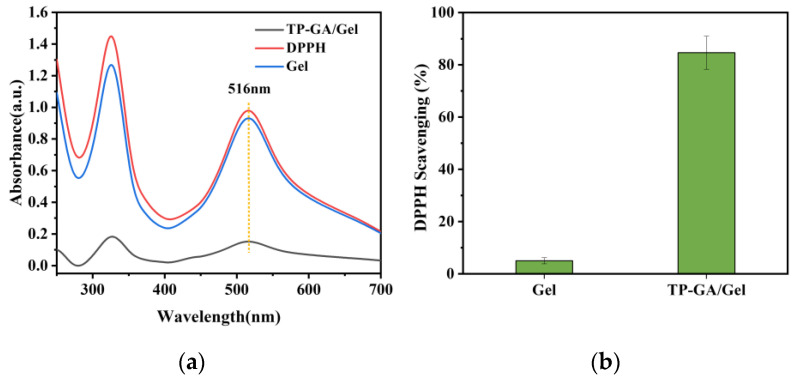
(**a**) UV absorption spectra of DPPH radicals by pure Gel and TP-GA/Gel adhesive; (**b**) scavenging rate of DPPH radicals by pure Gel and TP-GA/Gel adhesive.

TP exhibits antioxidant properties, and TP-GA/Gel adhesives are expected to possess similar characteristics. The classical DPPH assay was used to examine the free radical scavenging ability of TP-GA/Gel adhesives. As shown in Figure 7a, the absorbance of DPPH at 516 nm showed almost no decrease after treatment with pure Gel. However, a significant decrease in absorbance was observed after treatment with the TP-GA/Gel adhesive, resulting in a scavenging rate of 84.64%. This high DPPH radical scavenging rate was attributed to the abundant phenolic hydroxyl groups in the TP-GA/Gel adhesive, which donate hydrogen atoms to neutralize the unpaired electrons in DPPH radicals, forming DPPH-H and changing the DPPH solution from its original purple color to light yellow color [29]. These results indicate that TP-GA/Gel is an adhesive with strong antioxidant properties, making it beneficial for promoting skin repair.

### 3.5. Cytotoxicity Experiments Analysis

**Figure 8:**
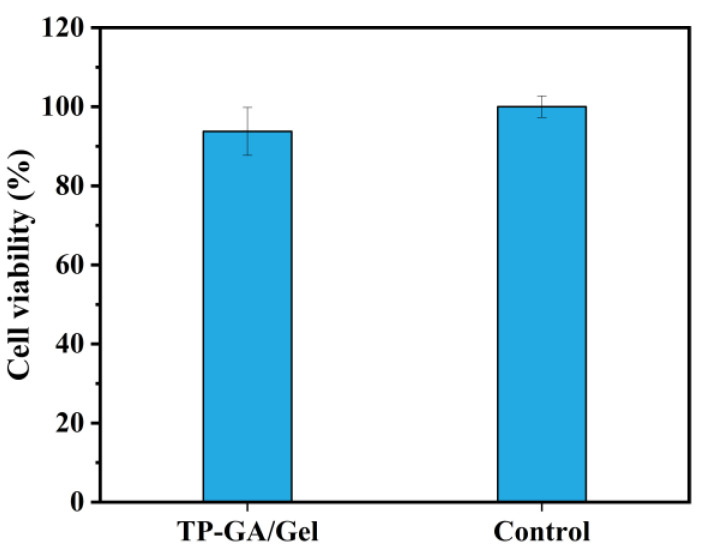
Survival of L929 cells cultured with TP-GA/Gel adhesive extract.

Mouse fibroblasts (L929) were used to evaluate the biocompatibility of the TP-GA/Gel adhesive. As observed under a light microscope (Figure S3), L929 cells cultured with the TP-GA/Gel adhesive extract showed nearly identical morphology to those in the control group, maintaining a complete, elongated spindle shape. The cell viability, measured using the CCK-8 assay, reached 93.76% (Figure 8), meeting the cytotoxicity class I criterion (cell viability greater than 75%) [30]. Since Gel, GA, and TP are all naturally derived compounds known for their good biocompatibility and low toxicity, they have less effect on cells. These results confirm that the TP-GA/Gel adhesive possesses excellent biocompatibility.

### 3.6. Wound Closure Evaluation

**Figure 9:**
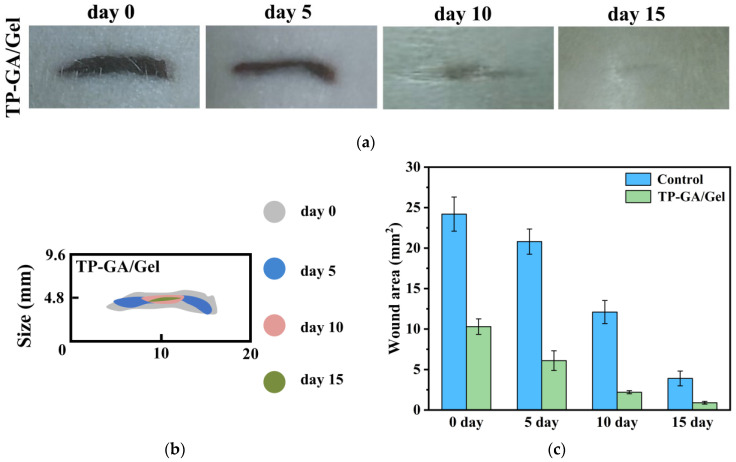
(**a**) Photographs of SD rat wounds on day 0, day 5, day 10, and day 15 of wound healing, respectively; (**b**) schematic diagram of simulated wound closure; and (**c**) remaining area of wounds on day 0, day 5, day 10, and day 15.

To investigate the potential of TP-GA/Gel adhesive in promoting wound healing, in vivo animal experiments were performed using SD rats. As depicted in Figure S4a and Figure 9a, live images of the blank control group and the TP-GA/Gel adhesive group were captured from day 0 to day 15 of wound healing. Both groups showed progressive wound healing, but the TP-GA/Gel adhesive group demonstrated significantly better healing. In the animal experiments, the adhesive remained intact without dissolving and left minimal residue after 15 days. Figure S4b and Figure 9b present a comparison of wound areas at different healing stages, clearly showing a reduction in wound size for both groups. However, the overall wound areas in rats treated with TP-GA/Gel adhesive were consistently smaller than those in the blank control group. Figure 9c quantifies the wound areas at each time point: in the blank control group, wound areas decreased from 24.2 mm^2^ on day 0 to 3.9 mm^2^ on day 15, whereas in the TP-GA/Gel adhesive group, wound areas reduced more significantly from 10.3 mm^2^ to 0.9 mm^2^ over the same period. Notably, the wound area was reduced by 57.43% on day 0, and by day 15, the wound was nearly scar-free, demonstrating that TP-GA/Gel adhesive greatly accelerates wound healing.

## 4. Conclusions

The widely available and cost-effective Gel was used as the matrix, and the TP-GA/Gel adhesive was synthesized by modifying the Gel protein molecular chains through the auto-oxidative polymerization of GA in solution, followed by the cross-linking of the Gel molecular chains using TP. The adhesive exhibited an obvious viscoelastic solid state with good mechanical strength. Unlike conventional cyanoacrylate adhesives, the TP-GA/Gel adhesive demonstrates excellent reproducible adhesion, allowing it to be reset on demand and reused multiple times. In addition, TP-GA/Gel possesses remarkable antimicrobial and antioxidant properties, along with good biocompatibility. In conclusion, TP-GA/Gel holds great potential for clinical applications and provides a novel approach to designing bio-based adhesives with outstanding adhesive properties and multiple reusability.

## Data Availability

The data presented in this study are available on request from the corresponding author.

## Supplementary Materials

The following supporting information can be downloaded at https://www.mdpi.com/article/10.3390/biomimetics10030149/s1: Figure S1: Antibacterial graph of TP-GA/Gel against *E. coli* (a) and *S. aureus* (b); Figure S2: SEM images of *E. coli* (a) and *S. aureus* (b) in the blank control group; Figure S3: Light microscopy of blank control and L929 cells cultured with TP-GA/Gel adhesive extract; and Figure S4: (a) Photographs of SD rat wounds on day 0, day 5, day 10, and day 15 of wound healing in the blank control group, respectively; (b) Schematic diagram of simulated wound closure in the blank control group.

## Abbreviations

The following abbreviations are used in this manuscript:

Gel	Gelatin
GA	Gallic acid
TP	Tea polyphenols
UV−vis	Ultraviolet−visible spectrophotometer
FT-IR	Fourier transform infrared (spectroscopy)
SEM	Scanning electron microscope
