# Supplementary material for: Biomimetic Design of Underwater Adhesives Based on Tea Polyphenol-Modified Gelatin
Source: Biomimetics (Basel). 2025 Feb 28;10(3):149. doi: 10.3390/biomimetics10030149 (PMC11940816; doi:10.3390/biomimetics10030149)
Supplement: Supplementary file 1 [file biomimetics-10-00149-s001.zip › biomimetics-3501611-supplementary.pdf]

Supporting Information

Biomimetic Design of Underwater Adhesives Based on Tea Polyphenol-Modified Gelatin

Ziwei Wu [1,2], Zhipeng Li [1,2], Yixiao Li [1,2], Haoyu Wang [1,2], Jiang Yue [3,*] and Tieling Xing [1,2,*]

[1] National Engineering Laboratory for Modern Silk, 20244215044@stu.suda.edu.cn (Z.W.); 20224215010@stu.suda.edu.cn (Z.L.); 20244215039@stu.suda.edu.cn (Y.L.); 20245215079@stu.suda.edu.cn (H.W.)
[2] China National Textile and Apparel Council Key Laboratory of Natural Dyes, College of Textile and Clothing Engineering, Soochow University, Suzhou 215123, China
[3] School of Medicine, Shanghai Jiaotong University, Shanghai 200127, China
*Correspondence: dragonfox@sjtu.edu.cn (J.Y.); xingtieling@suda.edu.cn (T.X.); Tel.: +86-512-6706-1175 (T.X.)

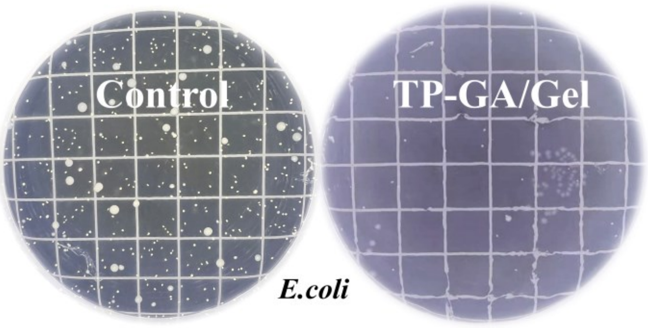

(**a**)

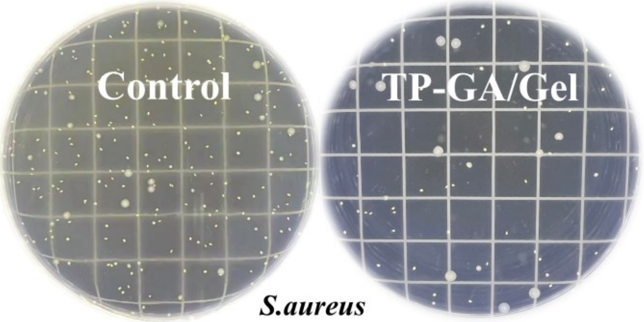

(**b**)

Figure S1. Antibacterial graph of TP-GA/Gel against *E. coli* (**a**) and *S. aureus* (**b**).

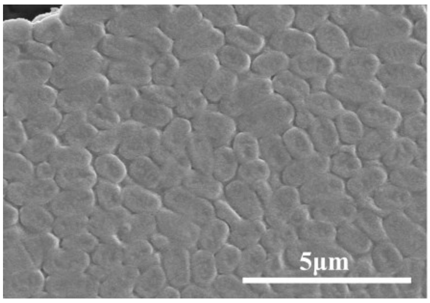

(**a**)

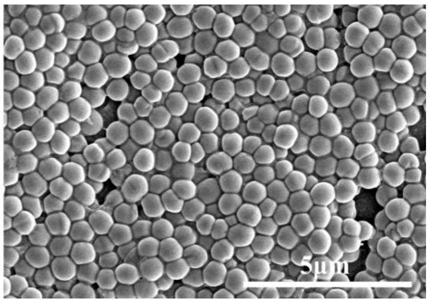

(**b**)

Figure S2. SEM images of *E. coli* (**a**) and *S. aureus* (**b**) in the blank control group.

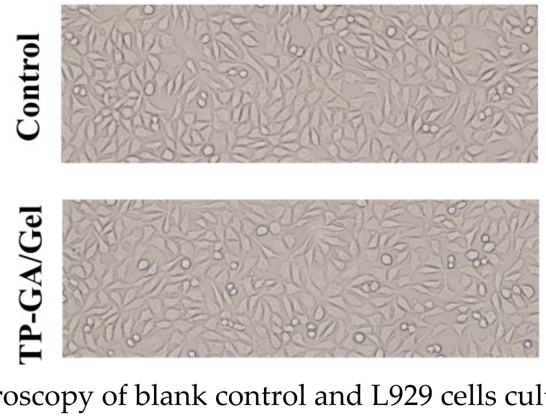

Figure S3. Light microscopy of blank control and L929 cells cultured with TP-GA/Gel adhesive extract.

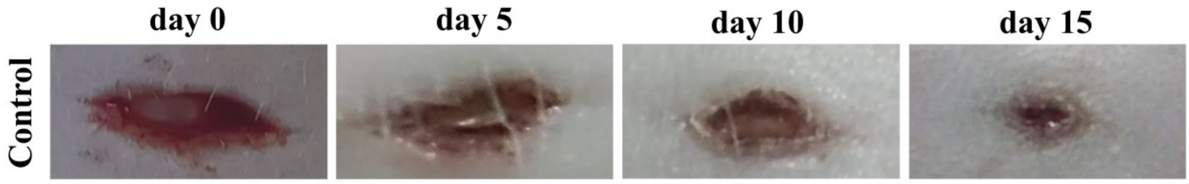

(**a**)

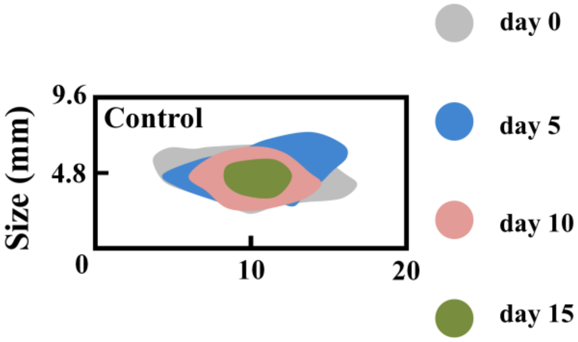

(**b**)

Figure S4. (**a**) Photographs of SD rat wounds on day 0, day 5, day 10, and day 15 of wound healing in the blank control group, respectively; (**b**) Schematic diagram of simulated wound closure in the blank control group.
